# Accuracy of Smartphone Camera Applications for Detecting Atrial Fibrillation: A Systematic Review and Meta-analysis

**DOI:** 10.1001/jamanetworkopen.2020.2064

**Published:** 2020-04-03

**Authors:** Jack W. O’Sullivan, Sam Grigg, William Crawford, Mintu P. Turakhia, Marco Perez, Erik Ingelsson, Matthew T. Wheeler, John P. A. Ioannidis, Euan A. Ashley

**Affiliations:** 1 Division of Cardiology, Department of Medicine, Stanford University School of Medicine, Stanford, California; 2 Meta-Research Innovation Center at Stanford (METRICS), Stanford University, Stanford, California; 3 Department of Medicine, University of Melbourne, Melbourne, Australia; 4 Oxford University Medical School, Oxford, United Kingdom; 5 Center for Digital Health, Stanford University School of Medicine, Stanford, California; 6 Veterans Affairs Palo Alto Health Care System, Palo Alto, California; 7 Stanford Cardiovascular Institute, Department of Medicine, Stanford University School of Medicine, Stanford, California; 8 Stanford Diabetes Research Center, Department of Medicine, Stanford University School of Medicine, Stanford, California; 9 Stanford Prevention Research Center, Department of Medicine, Stanford University School of Medicine, Stanford, California; 10 Department of Health Research and Policy, Stanford University School of Medicine, Stanford, California; 11 Department of Genetics, Stanford University School of Medicine, Stanford, California

## Abstract

**Importance:**

Atrial fibrillation (AF) affects more than 6 million people in the United States; however, much AF remains undiagnosed. Given that more than 265 million people in the United States own smartphones (>80% of the population), smartphone applications have been proposed for detecting AF, but the accuracy of these applications remains unclear.

**Objective:**

To determine the accuracy of smartphone camera applications that diagnose AF.

**Data Sources and Study Selection:**

MEDLINE and Embase were searched until January 2019 for studies that assessed the accuracy of any smartphone applications that use the smartphone’s camera to measure the amplitude and frequency of the user’s fingertip pulse to diagnose AF.

**Data Extraction and Synthesis:**

Bivariate random-effects meta-analyses were constructed to synthesize data. The study followed the Preferred Reporting Items for Systematic Reviews and Meta-analyses (PRISMA) of Diagnostic Test Accuracy Studies reporting guideline.

**Main Outcomes and Measures:**

Sensitivity and specificity were measured with bivariate random-effects meta-analysis. To simulate the use of these applications as a screening tool, the positive predictive value (PPV) and negative predictive value (NPV) for different population groups (ie, age ≥65 years and age ≥65 years with hypertension) were modeled. Lastly, the association of methodological limitations with outcomes were analyzed with sensitivity analyses and metaregressions.

**Results:**

A total of 10 primary diagnostic accuracy studies, with 3852 participants and 4 applications, were included. The oldest studies were published in 2016 (2 studies [20.0%]), while most studies (4 [40.0%]) were published in 2018. The applications analyzed the pulsewave signal for a mean (range) of 2 (1-5) minutes. The meta-analyzed sensitivity and specificity for all applications combined were 94.2% (95% CI, 92.2%-95.7%) and 95.8% (95% CI, 92.4%-97.7%), respectively. The PPV for smartphone camera applications detecting AF in an asymptomatic population aged 65 years and older was between 19.3% (95% CI, 19.2%-19.4%) and 37.5% (95% CI, 37.4%-37.6%), and the NPV was between 99.8% (95% CI, 99.83%-99.84%) and 99.9% (95% CI, 99.94%-99.95%). The PPV and NPV increased for individuals aged 65 years and older with hypertension (PPV, 20.5% [95% CI, 20.4%-20.6%] to 39.2% [95% CI, 39.1%-39.3%]; NPV, 99.8% [95% CI, 99.8%-99.8%] to 99.9% [95% CI, 99.9%-99.9%]). There were methodological limitations in a number of studies that did not appear to be associated with diagnostic performance, but this could not be definitively excluded given the sparsity of the data.

**Conclusions and Relevance:**

In this study, all smartphone camera applications had relatively high sensitivity and specificity. The modeled NPV was high for all analyses, but the PPV was modest, suggesting that using these applications in an asymptomatic population may generate a higher number of false-positive than true-positive results. Future research should address the accuracy of these applications when screening other high-risk population groups, their ability to help monitor chronic AF, and, ultimately, their associations with patient-important outcomes.

## Key Points

**Question:**

What is the overall accuracy of smartphone camera applications that diagnose and screen for atrial fibrillation (AF)?

**Findings:**

In this meta-analysis of 10 primary diagnostic accuracy studies with 3852 participants, all applications that used photoplethysmography signals to diagnose AF had high sensitivity and specificity. However, the modeled positive predictive value for screening an asymptomatic population aged 65 years and older with a history of hypertension was approximately 20% to 40%, although the negative predictive value was near 100%.

**Meaning:**

In this study, smartphone camera applications had high sensitivity and specificity for diagnosing AF and appeared adequate for ruling out AF, but their modest positive predictive value suggests that these devices will generate a higher number of false-positive than true-positive results.

## Teaser

This systematic review and meta-analysis assesses the accuracy of smartphone camera applications that diagnose atrial fibrillation among US adults aged 65 years and older.

## Introduction

Atrial fibrillation (AF) is the most common cardiac arrhythmia.^1^ In the United States, as many as 6 million individuals are estimated to have AF,^1,2,3^ with more than 12 million projected to be diagnosed by 2030.^1,4^ Atrial fibrillation causes substantial morbidity and mortality, predominantly because it results in a 5-fold increased risk of stroke.^5^

Despite its high prevalence, much AF remains undiagnosed (as many as 700 000 individuals in the United States),^6^ likely owing to the disease’s episodic nature and its propensity to remain asymptomatic.^7^ Undiagnosed AF is of particular concern given that approximately 20% of all AF-induced strokes occur among patients with undiagnosed AF,^8^ indicating that many of these strokes could have been prevented with appropriate treatment. Appropriate, early diagnosis is particularly pertinent for AF given that oral anticoagulants may have a favorable risk-benefit ratio for many patients.^9,10,11^

Because AF is highly prevalent,^1^ causes substantial morbidity,^5^ and is often undiagnosed,^6^ much debate has focused on screening.^12,13^ Although the science is still evolving, smart digital tools have emerged as a potential way to detect AF and monitor chronic AF.^14^ Given that more than 265 million people own smartphones in the United States (>80% of the population), smartphone camera applications have been proposed as accessible and widespread screening tools.^15,16^ These applications, which do not require any accessories, measure a user’s fingertip pulse via the camera and analyze the timing and morphology of a photoplethysmography (PPG) signal obtained via the pulse. Several studies have assessed the accuracy of smartphone camera applications for diagnosing AF, but all have been small, single studies. None have directly compared applications, and their results for single applications have been imprecise. We set out to determine the accuracy of smartphone camera applications that diagnose and screen for AF and to determine which application is the most accurate.

## Methods

### Protocol Registration and Study Design

The protocol for this systematic review was developed and registered a priori with PROSPERO (CRD42019125253).^17^ This systematic review and meta-analysis was conducted in line with the Preferred Reporting Items for Systematic Reviews and Meta-analyses (PRISMA) of Diagnostic Test Accuracy Studies reporting guideline.^18^

### Search Strategy and Eligibility Criteria

Using a structured search strategy (eAppendix 1 in the Supplement), we searched MEDLINE and Embase databases until January 31, 2019. We also searched unpublished trials on ClinicalTrials.gov and the World Health Organization International Clinical Trials Registry Platform. Furthermore, we searched the reference lists of included studies and similar articles in PubMed.

We included primary diagnostic accuracy studies that assessed the accuracy of any smartphone application that uses a smartphone camera to diagnose AF. We included studies in which diagnostic accuracy data were extractable (or able to be calculated), ie, that included the number of true-positives, true-negatives, false-positives, and false-negatives. We included studies of any smartphone application with human participants and used the Oxford English Dictionary definition of a *smartphone*, ie, “a mobile phone that performs many of the functions of a computer.”^19^ The devices had to be capable of diagnosing AF; we defined *capable* as the ability to produce at least a single lead electrocardiogram (ECG) trace, to analyze a heart rhythm tracing, and to make an AF diagnosis. For the primary analysis, we only included studies that used a reference standard of the ECG. To reach an AF diagnosis, these smartphone applications obtain a PPG signal from a user’s fingertip pulse via the camera. The regularity of the PPG signal is analyzed, both in terms of its morphology and its timing. A diagnosis of AF is made if the signal reaches a threshold of irregular rhythm (measured by root mean square of successive difference of R-R intervals, Shannon Entropy, and Poincare plots) and a consecutive period of nonidentical morphology (typically >30 seconds, measured in hertz) is observed. A full explanation of the methods is included in the eTable 1 in the Supplement.

We included conference papers and theses if we could extract accuracy data and the type of smartphone application used. We only included conference papers if their data did not overlap with published papers. We also excluded studies of smartwatches, handheld devices (including those that connect to smartphones, eg, AliveCor), smartphone applications that measure heart rate via other means than the camera, contactless smartphone heart rate monitors, conventional Holter monitors, and devices that exclusively measure heart rate or ECG intervals (eg, R-R interval, QT interval). We decided to exclude smartwatches because they are a different tool than smartphones, with continuous monitoring rather than ad hoc use. Moreover, the use of smartwatches is associated with a younger and more physically active population for largely different primary purposes. We were primarily interested in modeling screening among individuals aged 65 years and older, and there is evidence that approximately 40% of US residents older than 65 years own a smartphone (and approximately 60% of Americans aged 65-69 years own a smartphone),^20^ while only approximately 15% of those older than 50 years own a smartwatch.^21^ Moreover, when we designed the meta-analysis protocol, there was a dearth of data on smartwatches. Meta-analyses of a small number of studies are prone to bias and unreliable results,^22^ and we felt that the literature on smartwatches and AF was not ready for a formal meta-analysis.

### Study Screening and Data Extraction

Two of us (S.G. and W.C.) independently undertook a 3-step parallel review of article titles, abstracts, and full text (eAppendix 1 and eFigure 1 in the Supplement). Disagreements were resolved by a third author (J.W.O.).

Two authors (J.W.O. and S.G.) then extracted the following information: author, study design, algorithm used by the application, 2 × 2 table of diagnostic accuracy (ie, true-positives, false-positives, true-negatives, and false-negatives), type of smartphone, smartphone application, signs and symptoms of included patients, how and where participants were recruited, mean age of participants, reference standard, and time between smartphone camera recording and reference-standard arbitration.

### Quality Assessment

We used the Quality Assessment of Diagnostic Accuracy Studies 2 (QUADAS-2) tool to assess the risk of bias of included studies.^20^ This tool is the universally recommended risk-of-bias tool for assessing the methodological quality of diagnostic accuracy studies.^20^ The QUADAS-2 tool guides methodologists through an appraisal of the quality of studies that examine the accuracy of a certain test (ie, the index test) to diagnose a specific disease. The disease status of participants is defined by another, ideally criterion-standard test (ie, the reference test). The risk of bias of a diagnostic accuracy study is assessed across the 4 following domains: patient selection, the index test, the reference test, and flow and timing. The methodologist grades each domain as having a high, low, or unclear risk of bias. For our study, we appraised the methodological quality of all included studies. Risk-of-bias assessment allows for results to be stratified by primary study quality and provides the reader with insight into the methodologic robustness of the primary studies.

### Statistical Analysis

We constructed coupled forest plots with the sensitivity and specificity of all primary studies. These plots included studies of all smartphone applications, but different applications were labeled accordingly. Similarly, we labeled studies that used a non–criterion-standard reference test (1 study [10.0%]).

To synthesize data, we constructed bivariate random-effects meta-analyses to determine the meta-analyzed sensitivity and specificity.^21,22^ We then used hierarchical summary receiver operating characteristic (ROC) curve meta-analysis methods to construct summary ROC curves with accompanying 95% CIs for each smartphone application and for all applications collectively. From the meta-analyzed sensitivity and specificity, we calculated diagnostic odds ratios ([sensitivity × specificity]/[(1 – sensitivity) × (1 – specificity)]) for the applications meta-analyzed collectively and for each application individually.

We modeled the positive predictive values (PPVs) and negative predictive values (NPVs) for each application individually and for all applications collectively. To model PPV and NPV, we used the sensitivity and specificity calculated from our bivariate meta-analysis, the estimated prevalence of undiagnosed AF in the United States, and the total US population. We also extracted an estimate of the prevalence of hypertension in those aged 65 years and older with AF.^2,23^ We extracted the estimated prevalence measures of undiagnosed AF in people aged 65 years and older from 2 previous studies^6,24^ (ie, 1.3% and 3.2%). We then modeled the PPV and NPV for the 2 following population groups: individuals aged 65 years and older and individuals aged 65 years and older with a history of diagnosed hypertension (eAppendix 1 in the Supplement). We chose to perform this last analysis given that all participants aged 65 years and older with a history of hypertension would have actionable changes to their management if AF were detected; they would be eligible for anticoagulation medication according to both the American and European guidelines because their CHA_2_DS_2_-VASc score would be 2.^9,10^ The CHA_2_DS_2_-VASc score is is calculated according to a point system in which 2 points are assigned for a history of stroke or transient ischemic attack (S_2_) or age (A_2_) older than 75 years and 1 point each is assigned for age (A) of 65 to 74 years or a history of congestive heart failure (C), hypertension (H), diabetes (D), vascular disease (V; ie, myocardial infarction and peripheral artery disease), and female sex (sex category, Sc).

We performed several sensitivity analyses. These included sensitivity analyses to examine potential biases in the meta-analysis estimates as well as additional sensitivity analyses to examine potential biases in the modeled PPV and NPV.

For the sensitivity analyses focused on the meta-analysis, we performed the following analyses: (1) including the 1 study that used a non–criterion-standard reference test; (2) excluding the studies subject to verification bias^25^; (3) excluding studies in which the index and reference standard were not performed simultaneously or immediately after each other; (4) stratifying included studies by study design (ie, case-control vs cohort); (5) excluding studies with at least 1 domain rated as high risk of bias from QUADAS-2; and (6) excluding conference abstracts and theses, which was a post hoc sensitivity analysis conducted in line with a reviewer comment.

Additional sensitivity analyses had been prespecified^17^ but lacked sufficient data. Metaregressions were also performed to assess whether identified biases were associated with the results but should be interpreted with caution given the limited number of participants included in the metaregression analyses.

The additional sensitivity analyses focused on the modeling of PPV and NPV. In addition to the primary analysis (in which we used prevalence estimates of undiagnosed AF of 1.3%^6^ and 3.2%^24^), we conducted a sensitivity analysis using the prevalence of AF in the United States derived from the American Heart Association (AHA) Heart Disease and Stroke Statistics—2019 Update.^1^ The AHA estimated that the prevalence of AF in the United States was between approximately 2.7 million and 6.1 million individuals in 2010,^1^ an estimation that was also endorsed by the US Preventive Services Task Force (USPSTF).^26^ However, these estimates included the number of people who already have AF, whereas the estimates used in our primary model were estimates of undiagnosed AF. Given that we wanted to model PPVs and NPVs in a screening scenario, this AHA prevalence estimate was likely to inflate the PPV and NPV values compared with prevalence estimates of undiagnosed AF. Thus, our primary PPV and NPV model used the estimates of undiagnosed AF, but we also conducted a secondary analysis using the AHA/USPSTF prevalence estimates. We included sensitivity analyses using the AHA estimates for 2 reasons. First, the analyses best display the variation in AF prevalence among age groups, which has implications for the PPV and NPV at different ages. Second, the USPSTF endorsed the AHA AF prevalence estimates; given that an aim of this study was to address AF screening, we felt many policy makers would expect and possibly benefit from diagnostic accuracy measures based on USPSTF-endorsed AHA prevalence estimates.

Furthermore, the primary studies we included did provide AF prevalence estimates; however, we chose not to model PPV and NPV from these estimates for the 3 following reasons: (1) as we anticipated a priori, most included studies were case-control designs, which, by definition, lead to much greater prevalence estimates than would be expected in a screening population; (2) the disease prevalence estimates from included studies came from many different countries, which may not reflect US prevalence estimates; and (3) the included studies were a select and small sample of participants, which could upwardly bias prevalence estimates, which would then upwardly bias PPV estimates.

All analyses were conducted in R version 3.5.1 (R Project for Statistical Computing) using the libraries *mada* and *epitests*. For metaregressions, statistical significance was set at *P* < .05, and all tests were 2-tailed.

## Results

### Study Selection and Characteristics

We included 10 primary diagnostic accuracy studies^15,16,27,28,29,30,31,32,33,34^ from 1156 references identified from our database search. The oldest studies were published in 2016 (2 studies [20.0%]), while most studies (4 [40.0%]) were published in 2018. The included studies examined diagnostic test accuracy among 3852 participants, 934 (24.2%) of whom had AF. All included studies used ECG as their reference standard, except 1 study,^27^ which was excluded from the primary analysis, as described in the Methods section. The applications analyzed the pulsewave signal for a mean (range) of 2 (1-5) minutes. Three studies (30.0%) examined the accuracy of the FibriCheck application (Qompium),^31,33,34^ 3 (30.0%) assessed the Cardiio Rhythm Mobile application (Cardiio, Inc),^28,29,30^ 3 (30.0%) examined the Preventicus application (Preventicus),^15,27,32^ and 1 (10.0%) appraised a study version of the PULSE-SMART application^16^ (not currently commercially available). All studies were conducted using iPhones (or the device was not explicitly stated), and each application used similar underlying statistical and machine learning approaches for appraising pulse rate via pulse PPG. Table 1 outlines the key characteristics of the included studies (eTable 2 in the Supplement).^35^

**Table 1.** Characteristics of Included Studies

Source	Design	Application	Participants, No.	AF, No. (%)	Age, mean (SD), y	Hypertension, No. (%)
Brasier et al,^15^ 2019	Case-control	Preventicus	592	248 (41.9)	Median, 78	427 (72.1)
McManus et al,^16^ 2016	Case-control, before and after cardioversion	PULSE-SMART	121	104 (86.0)	65.9 (12.2)	AF group, 70 (71.4); SR group, 63 (69.2)
Krivoshei et al, ^27^ 2017	Case-control	Preventicus^a^	80	40 (50.0)	80 (8)	Not stated
Rozen et al,^28^ 2018	Case-control, before and after cardioversion	Cardiio Rhythm	98	96 (98.0)	67.7 (10.5)	Not stated
Yan et al,^29^ 2018	Cohort, patients recruited from cardiology ward	Cardiio Rhythm	217	75 (34.6)	70.3 (13.9)	130 (59.9)
Chan et al,^30^ 2016	Cohort	Cardiio Rhythm	1014	28 (2.8)	68.4 (12.2)	916 (90.4)
Grieten et al,^31^ 2018^b^	Cohort	FibriCheck	1056	8 (0.8)	59 (15)	Not stated
Karim et al,^32^ 2017^b^	Case-control	Preventicus	140	70 (50.0)	AF group, 74 (12); SR group, 60 (20)	Not stated
Vandenberk et al,^33^ 2018^b^	Cohort, patients with a history of AF recruited	FibriCheck	344	173 (50.3)	Not stated	Not stated
Mortelmans et al,^34^ 2017^c^	Case-control	FibriCheck	190	92 (48.4)	78 (8)	198 (83.5)

Abbreviations: AF, atrial fibrillation; SR, sinus rhythm.

^a^ We assumed this study tested the Preventicus application because the authors used the same algorithm as the other Preventicus studies, had the same authors, and cited the same methodological paper.^35^ Regardless, this study was excluded from the primary analysis because it used an imperfect reference standard.

^b^ Conference abstract.

^c^ Thesis for MSc degree.

### Sensitivity and Specificity

**Figure 1.**
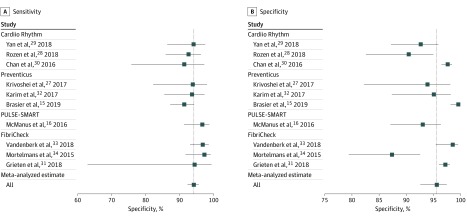
Sensitivity and Specificity for Each Study and the Overall Meta-analyzed Sensitivity and Specificity Boxes represent the point estimate, and whiskers are 95% CIs. The vertical line indicates the overall meta-analyzed point estimate.

**Figure 2.**
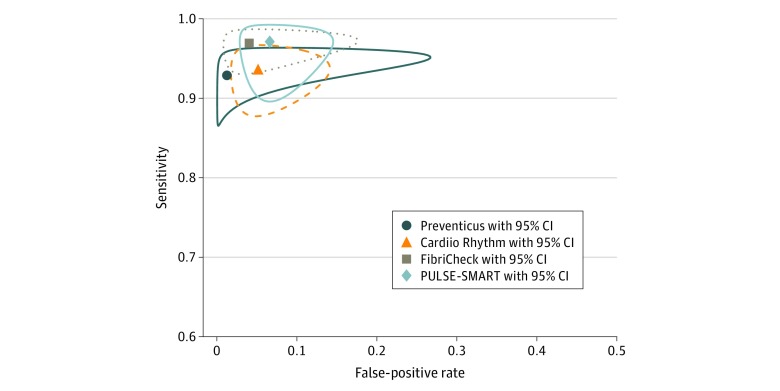
Summary Receiver Operating Characteristic Curve of the Meta-analyzed Sensitivity and Specificity for Smartphone Camera Applications

Table 2 and Figure 1 show the meta-analyzed sensitivity and specificity for each individual application and overall. The meta-analyzed sensitivity and specificity for all applications combined were 94.2% (95% CI, 92.2%-95.7%) and 95.8% (95% CI, 92.4%-97.7%), respectively. The sensitivity and specificity were high across all applications; sensitivity ranged from 92.9% (95% CI, 88.1%-95.8%) to 97.1% (95% CI, 91.4%-99.1%) and specificity, from 93.4% (95% CI, 87.3%-96.7%) to 98.7% (84.3%-99.9%). Figure 2 reports the summary ROC curves for each application; eFigure 6 in the Supplement reports the summary ROC curves for all applications combined. The diagnostic odds ratio for all studies was 400.5 (95% CI, 204.8-783.2) (eTable 3 in the Supplement).

**Table 2.** Meta-analyzed Sensitivity and Specificity for Applications Collectively and Individually

Application	Score, % (95% CI)	
	Sensitivity	Specificity
Cardiio Rhythm mobile	93.5 (89.2-96.2)	94.8 (88.3-97.8)
FibriCheck	96.9 (94.1-98.4)	96.0 (86.6-98.9)
Preventicus^a^	92.9 (88.1-95.8)	98.7 (84.3-99.9)
PULSE-SMART^b^	97.1 (91.4-99.1)	93.4 (87.3-96.7)
All^a^	94.2 (92.2-95.7)	95.8 (92.4-97.7)

^a^ This analysis does not include the data from Krivoshei et al,^27^ which used an imperfect reference standard.

^b^ Only 1 study used this application, so the presented sensitivity and specificity were not meta-analyzed. The rest of the data are the meta-analyzed estimates.

### PPV and NPV

**Figure 3.**
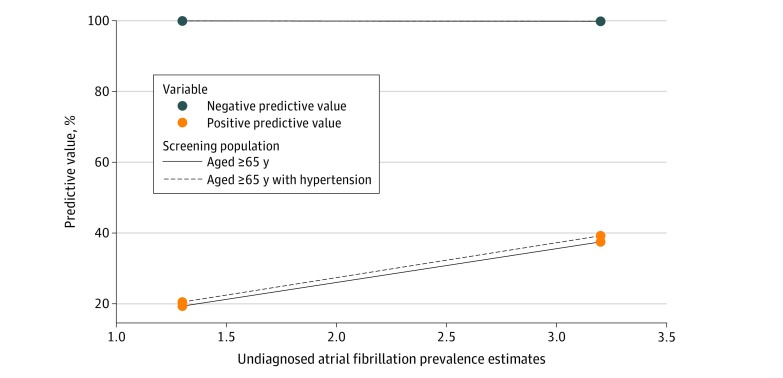
Positive and Negative Predictive Values for the Applications Collectively Among Different Population Groups

Figure 3 displays the modeled PPV and NPV for the applications collectively; eFigure 2 and eFigure 3 in the Supplement show the applications individually. For individuals aged 65 years and older, the meta-analyzed estimates of PPV and NPV for all applications for the detection of undiagnosed AF were 19.3% (95% CI, 19.2%-19.4%) and 99.9% (95% CI, 99.94%-99.95%), respectively, assuming an undiagnosed AF prevalence of 1.3%. With a prevalence estimate of 3.2% among the same population group, the PPV increased to 37.5% (95% CI, 37.4%-37.6%) but the NPV was essentially unchanged (99.8% [95% CI, 99.83%-99.84%]). The association between the different prevalence levels was not examined statistically. The PPV and NPV were higher when we examined individuals aged 65 years and older with hypertension. We found the PPV and NPV were 20.5% (95% CI, 20.4%-20.6%) and 99.9% (95% CI, 99.9%-99.9%), respectively, assuming a prevalence of 1.3%, and 39.2% (95% CI, 39.1%-39.3%) and 99.8% (95% CI, 99.8%-99.8%), respectively, assuming a prevalence of 3.2%. The full PPV and NPV estimates under different assumptions are reported in eTable 4 in the Supplement. From our model, we observed a PPV that varied from 13.7% to 44.3% for the studied applications (for the population aged ≥65 years, with a prevalence estimate of 1.3%), while the NPV remained essentially unchanged for each application (>99.8%). Using the undiagnosed AF prevalence of 3.2%, the PPV varied from 28.4% to 46.1%, while the NPV remained greater than 99.8% for all applications. Full details appear in eTable 4 in the Supplement, including the results for the group aged 65 years and older with hypertension.

### Quality Assessment

The QUADAS-2 assessment for all included studies appears in eTable 5 in the Supplement. No studies were rated as having a low risk of bias across all 4 domains. However, no studies were rated as high or unclear risk of bias across all domains. We conducted a number of sensitivity analyses that explored the associations of methodological variation with our results, including investigating the associations of verification bias, which occurred in 2 studies.^30,31^ Two studies^15,34^ excluded results based on PPG signal quality (7.3% for 1 study^15^ and 21.5% for the other study^34^); we did not include excluded data referring to poor PPG signal quality because it was not clear if these data should be classified as false-positives, true-positives, false-negatives, or true-negatives. However, our QUADAS-2 assessments reflect this potential bias, which was then captured in our sensitivity analyses. The number of non-AF arrhythmias detected by the application was reported in most studies; the proportion of these arrhythmias was generally low (median [range], 5.3% [1.2%-13.3%]).

### Sensitivity Analyses

The sensitivity analyses focused on the meta-analysis revealed an equivocal association with diagnostic performance (eTable 3 and eTable 6 in the Supplement). Metaregression models showed nonsignificant decreases in sensitivity for all sensitivity analyses that excluded studies with potential biases and a nonsignificant difference in sensitivity in case-control studies compared with cohort studies (eTable 7 in the Supplement). It is plausible that most of these analyses were limited by lack of power, and thus, we cannot definitively rule out an association of methodological limitations with our results. The results of the sensitivity analyses are described in detail in eAppendix 2 in the Supplement, and eTable 3 and eTables 6 to 8 in the Supplement report the quantitative results.

The sensitivity analysis for the modeled PPV and NPV showed a much higher PPV (58.3% for a prevalence estimate of 2.7 million and 77.4% for a prevalence estimate of 6.1 million). Furthermore, it showed an increasing PPV with an increased screening cutoff age (eg, 18 years compared with 65 years). The full results from this sensitivity analysis are reported in eFigure 4, eFigure 5, and eTable 4 in the Supplement.

## Discussion

We assessed the accuracy of smartphone camera applications for detecting AF. We found that all smartphone camera applications individually had a high sensitivity and specificity, and this remained true for the meta-analyzed estimate for all applications collectively. We estimated that this would translate to a modest PPV and near-perfect NPV in population settings with substantial burden of AF, eg, people older than 65 years with hypertension. Most studies had methodological limitations.

Smart devices have been proposed to help to detect undiagnosed AF and aid the management of patients with known AF.^36^ Regarding AF screening, if any of these applications reach a diagnosis of sinus rhythm in a healthy, asymptomatic person, it is likely this person does not have AF (ie, true-negative). Conversely, we cannot draw the same conclusion from a positive result. In fact, our model suggests that if these applications detect AF in an asymptomatic person, the result is most likely to be a false-positive. From these results, it would appear premature to use these devices among healthy individuals or to use them to screen an asymptomatic population, including those older than 65 years with hypertension. It would be interesting to examine whether the PPV would improve if these applications were used to screen a further selective high-risk population, for instance among patients with a CHA_2_DS_2_-VASc score of at least 3, those at high genetic risk, or those with chronic AF. However, downstream consequences of early detection would need to be carefully considered along with sufficient data on the magnitude of overdiagnosis as well as the downstream diagnostic and treatment burden.^37,38,39^

There seemed to be 3 clinical situations in which these applications could be used (or are currently being used), as follows: as a screening tool, as a diagnostic test, and/or as a monitoring device. First, it is plausible that people without symptoms but who are concerned about their risk of AF may use these applications to ascertain whether or not they have the disease (ie, screening test). We anticipated this use and modeled the PPV and NPV for a population for whom the detection of AF would lead to an immediate change in their management (those aged ≥65 years with hypertension, ie, with a CHA_2_DS_2_-VASc score of 2). Second, people who have symptoms that may be suggestive of AF may use these smartphone camera applications to try and self-diagnose AF (ie, diagnostic test). We do not necessarily approve of this use, but these applications are currently commercially available, cheap to download, and marketed to be used as such. Our results have provided both users and health care professionals who care for these patients the diagnostic accuracy of these applications collectively and individually for the commercially available applications. Third, we believe it is plausible that patients with an established diagnosis of paroxysmal AF may use these smartphone camera applications to monitor for relapses of AF (ie, monitoring device). Patients with paroxysmal AF alternate between sinus rhythm and AF. When these patients develop symptoms suggestive of AF, many are instructed to take their pulse and try to self-diagnose a relapse of AF. A confirmed relapse of AF may lead some patients to take rate-controlling medication or to seek medical attention. Some patients may seek to aid self-assessment of their pulse with a more objective measure, eg, a smartphone camera application.

Our results do not directly address another proposed use of these applications, which is chronic disease management. Patients with AF often alternate between sinus rhythm and AF; the ability for patients to self-diagnose when they are in AF may enable them to seek (or not seek) medical care as appropriate. Although we were not able to formally model the PPV for a population monitoring AF, it is plausible that it would be higher than what we observed for the screening population (given that those with paroxysmal AF have a far greater pretest probability than an asymptomatic population that is being screened for AF). Future research addressing this unknown would be advantageous.

Besides smartphone applications, there have been a number of studies that investigated the diagnostic accuracy of other smart devices, most notably, the Apple Heart Study.^40^ The Apple Heart Study differed from our study given that it recruited participants from a real-world setting, recruited a larger number of participants, used a different method and unit of analysis to calculate PPV and NPV (ie, tachograms rather than participants), and used a smartwatch that could passively measure PPG signals (ie, heart rate). Given the numerous differences between the primary studies included in our meta-analysis and the Apple Heart Study, a comparison of accuracy metrics and the respective inferences about PPV are unlikely to be robust. However, there have also been a number of studies conducted on AliveCor.^41^ The AliveCor accessory is a stand-alone device rather than a smartphone application that uses the smartphone’s camera. Previous studies have found the AliveCor accessory to have a range of sensitivities and specificities from 95% to 100% and 94% to 99%.^15,42,43,44,45^

The outstanding research question that needs to be addressed before any form of AF screening is implemented is that of the treatment of asymptomatic AF captured through screening. Most randomized clinical trials (RCTs) that examined the effect of anticoagulation medication on stroke risk among patients with AF were conducted on individuals with an established diagnosis of AF.^11^ Thus, it remains unclear whether patients with AF uncovered via screening would benefit from receiving anticoagulation medication. It is likely that the amount of benefit derived from anticoagulation medication will be related to an individual’s burden of AF and their CHA_2_DS_2_-VASc score; thus, stratification of results via these variables would be most useful to aid clinical decisions. Similarly, it would be of value if future diagnostic accuracy research stratified their results by the burden of AF and CHA_2_DS_2_-VASc scores, ie, if PPV and NPV were presented by, for instance, the duration of AF and by each respective CHA_2_DS_2_-VASc score.

Aside from RCTs addressing the treatment of asymptomatic AF, it would also be valuable for smart diagnostic tools to be examined in RCTs, eg, smartphone camera applications compared with conventional diagnostic tools (eg, ECG) and/or other novel smart diagnostic tools.^46^ These RCTs could determine the effect of these tools in terms of accuracy (eg, sensitivity and specificity) as well as important clinical outcomes (eg, the number of strokes among patients whose diagnosis and treatment were guided by a smartphone camera application vs ECG). Furthermore, once more data are generated, a meta-analysis examining the AF-related performance of different smartwatches would also be valuable.

We were able to model PPV and NPV to simulate the use of these applications as screening tools. However, we were not able to model how these devices would perform to help to monitor chronic, paroxysmal AF. Future research addressing this unknown would be valuable. It would also be valuable if future research addressed the value of repeat screening and at what interval.

Lastly, we do not endorse any application or application company and believe future studies will aid in distinguishing which, if any, applications or algorithms perform better than others. Future diagnostic accuracy studies should avoid the methodological flaws we described and include all PPG signals regardless of signal quality.

### Strengths and Limitations

The strengths of systematic reviews have been well-described.^47^ Briefly, they capture all the available evidence on a topic, increase the sample size of the analysis, and can thus facilitate a more precise result. We presented a systematic review of smartphone camera applications for diagnosing AF and included nearly 4000 participants in our meta-analysis, nearly 4-fold more than the largest single primary study. A further strength of our study is the extrapolation of our results beyond a meta-analysis. We also calculated the PPV and NPV, using up-to-date estimates of undiagnosed AF prevalence in the United States and US population estimates. The PPV and NPV estimates are particularly useful for clinicians and health policy workers, given that they differ substantially from the sensitivity and specificity and better reflect the clinical utility of these applications. The presentation of PPV and NPV stratified by different high-risk patient groups is also likely to be useful for clinicians and policy makers. Furthermore, our sensitivity analysis can be considered a strength. We identified numerous biases present in the included studies and quantitatively assessed their associations with our results via metaregression and used 4 different AF prevalence estimates in our model to calculate the PPV and NPV.

Our study has limitations. First, all studies had some methodological limitations or were not adequately reported. We anticipated this and explored the associations of this methodological variation with outcomes. We found that none of the methodological flaws we identified had a significant association with the results, but most of these analyses were likely restricted by low power. Nevertheless, we highlighted methodological flaws that should be avoided in future studies. Second, most of the included studies were funded or completed by manufacturers of the various applications. Future, independent research, especially RCTs, would be welcome. Third, it is unknown how many studies on this topic remain unpublished given that this is not a field where registration would be enforced. We cannot exclude the possibility that studies with unfavorable results have remained unpublished or that some of the reported 2 × 2 tables were not selectively reported. Fourth, a substantial proportion of the included studies are recent, and their data have been presented in conferences but are not yet peer reviewed. Thus, extra caution is required. Fifth, it is plausible that the PPV for the population group aged 65 and older with hypertension is higher than we report given that much hypertension in the elderly population is undiagnosed.^48^ Sixth, a model can never replace real-world data; it is possible that a study using real-world data would find substantially different PPV and NPV than we reported. Seventh, we were unable to model the PPV and NPV for patients with CHA_2_DS_2_-VASc scores greater than 2 because we could not obtain reliable estimates of heart failure, diabetes, stroke, and peripheral vascular disease. Eighth, we used 2 prevalence estimates for undetected AF. These estimates varied modestly (ie, 1.3% vs 3.2%), but both were derived from robust, well-conducted studies. However, it is plausible that the true prevalence of undetected AF lies somewhere between these estimates, and thus, the true PPV lies somewhere between the respective estimates (ie, approximately 20% to approximately 40%). Ninth, it is plausible that smartphone use is lower in those aged 65 years and older; however, recent evidence suggests that approximately 40% of US residents aged 65 years and older own and use smartphones and that use is increasing at a “record rate.”^49^ Also importantly, in the population group that is more likely to benefit from AF screening (ie, aged 65-69 years), approximately 60% own a smartphone in the United States.^49^ Tenth, included primary studies did not stratify their results by ethnicity or race; it remains unclear if the accuracy of smartphone camera applications varies by race/ethnicity.

## Conclusions

In this study, all smartphone camera applications had a relatively high sensitivity and specificity. These applications seem to be able to rule out AF in a healthy, asymptomatic patient; however, a positive result appears more likely to be a false-positive than a true-positive.

## Notes

Supplement. **eAppendix 1.** Search Strategy and Positive and Negative Predictive Values **eAppendix 2.** Sensitivity Analyses **eFigure 1. **Study Flow Diagram **eFigure 2. **PPV and NPV of All Specific Applications Using Undiagnosed AF Prevalence Estimate of 1.3% **eFigure 3. **PPV and NPV of All Specific Applications Using Undiagnosed AF Prevalence Estimate of 1.3% and 3.2% Among Individuals Aged 65 Years and Older With Hypertension **eFigure 4. **PPV and NPV for Each Application Using the AHA AF Prevalence Estimates **eFigure 5. **PPV for All Age Groups Using AHA AF Prevalence Estimates **eFigure 6. **Summary Receiver Operating Characteristic Curve for Meta-analyzed Sensitivity and Specificity for All Applications Combined **eTable 1. **Algorithm Details for Each Application **eTable 2. **Extended Characteristics of Included Studies **eTable 3. **Diagnostic Odds Ratio **eTable 4. **PPV and NPV **eTable 5. **Full QUADAS-2 Assessment **eTable 6. **Meta-analyzed Sensitivities and Specificities **eTable 7. **Metaregression **eTable 8. **Meta-analyzed Sensitivity and Specificity for Individual Applications **eReferences**
