# Supplementary material for: Accuracy of Smartphone Camera Applications for Detecting Atrial Fibrillation: A Systematic Review and Meta-analysis
Source: JAMA Netw Open. 2020 Apr 3;3(4):e202064. doi: 10.1001/jamanetworkopen.2020.2064 (PMC7125433; doi:10.1001/jamanetworkopen.2020.2064)
Supplement: Supplement. — eAppendix 1. Search Strategy and Positive and Negative Predictive Values eAppendix 2. Sensitivity Analyses eFigure 1. Study Flow Diagram eFigure 2. PPV and NPV of All Specific Applications Using Undiagnosed AF Prevalence Estimate of 1.3% eFigure 3. PPV and NPV of All Specific Applications Using Undiagnosed AF Prevalence Estimate of 1.3% and 3.2% Among Individuals Aged 65 Years and Older With Hypertension eFigure 4. PPV and NPV for Each Application Using the AHA AF Prevalence Estimates eFigure 5. PPV for All Age Groups Using AHA AF Prevalence Estimates eFigure 6. Summary Receiver Operating Characteristic Curve for Meta-analyzed Sensitivity and Specificity for All Applications Combined eTable 1. Algorithm Details for Each Application eTable 2. Extended Characteristics of Included Studies eTable 3. Diagnostic Odds Ratio eTable 4. PPV and NPV eTable 5. Full QUADAS-2 Assessment eTable 6. Meta-analyzed Sensitivities and Specificities eTable 7. Metaregression eTable 8. Meta-analyzed Sensitivity and Specificity for Individual Applications eReferences [file jamanetwopen-3-e202064-s001.pdf]

Supplementary Online Content

O'Sullivan JW, Grigg S, Crawford W, et al. Accuracy of smartphone camera applications for detecting atrial fibrillation: a systematic review and meta-analysis. *JAMA Netw Open*. 2020;3(4):e202064. doi:10.1001/jamanetworkopen.2020.2064

eAppendix 1. Search Strategy and Positive and Negative Predictive Values
eAppendix 2. Sensitivity Analyses
eFigure 1. Study Flow Diagram
eFigure 2. PPV and NPV of All Specific Applications Using Undiagnosed AF Prevalence Estimate of 1.3%
eFigure 3. PPV and NPV of All Specific Applications Using Undiagnosed AF Prevalence Estimate of 1.3% and 3.2% Among Individuals Aged 65 Years and Older With Hypertension
eFigure 4. PPV and NPV for Each Application Using the AHA AF Prevalence Estimates
eFigure 5. PPV for All Age Groups Using AHA AF Prevalence Estimates
eFigure 6. Summary Receiver Operating Characteristic Curve for Meta-analyzed Sensitivity and Specificity for All Applications Combined
eTable 1. Algorithm Details for Each Application
eTable 2. Extended Characteristics of Included Studies
eTable 3. Diagnostic Odds Ratio
eTable 4. PPV and NPV
eTable 5. Full QUADAS-2 Assessment
eTable 6. Meta-analyzed Sensitivities and Specificities
eTable 7. Metaregression
eTable 8. Meta-analyzed Sensitivity and Specificity for Individual Applications
eReferences

This supplementary material has been provided by the authors to give readers additional information about their work.

© 2020 O'Sullivan JW et al. *JAMA Network Open.*

eAppendix 1. Search Strategy and Positive and Negative Predictive Values

Search strategy

Our search strategy for MEDLINE is as follows:
"Smartphone"[Mh] OR "smartphone camera"[tw] OR "mobile phone"[tw] OR "mobilephone"[tw] OR "Pulse Wave Analysis"[Mh] OR "Photoplethysmography"[Mh] OR "PPG"[tw] OR "photoplethysmograph*"[tw] OR "cardiio"[tw] OR "Cardiio Rhythm"[tw] OR "FibriCheck"[tw] OR "Qompium"[tw] OR "Cardiio Rhythm Mobile Application"[tw] OR "CRMA"[tw] OR "Photo AFib Detector"[tw] OR "cardiac diagnosis"[tw] OR "preventicus"[tw] OR "iPhone"[tw] OR "samsung"[tw] OR "apple"[tw] OR "huawei"[tw] OR "oppo"[tw] OR "google pixel"[tw]) AND ("Atrial Fibrillation"[Mh] OR "Atrial Flutter"[Mh] OR "AF"[tw] OR "atrial"[tw] OR "Atrial Fibrillation"[tw] OR "Atrial Flutter"[tw] OR "AFib"[tw])

Our search strategy for EMBASE is as follows:
('iphone*':ti,ab,kw OR 'smartphone*':ti,ab,kw OR 'mobile phone*':ti,ab,kw OR 'mobile ecg':ti,ab,kw OR 'cell phone*':ti,ab,kw OR 'mobile application*':ti,ab,kw OR 'pulse wave analysis':ti,ab,kw OR 'photoplethysmography':ti,ab,kw OR 'ppg':ti,ab,kw OR 'photoplethysmograph*':ti,ab,kw OR 'cardiio':ti,ab,kw OR 'cardiio rhythm':ti,ab,kw OR 'fibricheck':ti,ab,kw OR 'qompium':ti,ab,kw OR 'cardiio rhythm mobile application':ti,ab,kw OR 'crma':ti,ab,kw OR 'photo afib detector':ti,ab,kw OR 'preventicus':ti,ab,kw OR 'iphone':ti,ab,kw OR 'samsung':ti,ab,kw OR 'apple':ti,ab,kw OR 'huawei':ti,ab,kw OR 'oppo':ti,ab,kw OR 'google pixel':ti,ab,kw) AND ('af':ti,ab,kw OR 'atrial':ti,ab,kw OR 'atrial fibrillation':ti,ab,kw OR 'atrial flutter':ti,ab,kw)

Positive and Negative Predictive Values

For these analyses, we extracted published data on a) AFib USA prevalence, b) the total USA population (including breakdowns of population by age-group for certain analyses) and for two analyses c.1) the prevalence of hypertension in the US population and the c.2) prevalence of people with AFib who also have hypertension. The below table reports the data we extracted and the source it comes from. For the secondary analyses using the AHA AFib prevalence estimates, 2010 census data was used for the total US population because the estimate of USA AFib prevalence from the AHA and USPTF is from 2010 (note this is a secondary analysis).

Primary analysis

**Metric**	**Data**	**Source**

© 2020 O’Sullivan JW et al. *JAMA Network Open.*

USA AFib Prevalence	1.3%	Mintu P. Turakhia , Jason Shafrin, Katalin Bognar, Jeffrey Trocio, Younos Abdulsattar, Daniel Wiederkehr, Dana P. Goldman. Estimated prevalence of undiagnosed atrial fibrillation in the United States. *PLOS One. 2018 Apr 12;13(4):e0195088*[1]
	3.2%	The Apple Heart Study[2]
USA AFib Prevalence in those aged ≥ 65 years	1.3% * 0.82	82% of AFib occurs in participants aged ≥ 65[3]
	3.2% * 0.82	
The prevalence of AFib in those aged over 65 and with hypertension.	(1.3% * 0.82)*0.84	As stated above, 82% of AFib occurs in those aged ≥ 65[3] and 84% of those with AFib have hypertension[4]
	(3.2% * 0.82)*0.84	
Total US population over the age of 65 and with a history of hypertension	40,267,984*0.782	40,267,984 is the number of people aged ≥ 65 in 2010 US census.[7] 78.2% of 2 65 have hypertension.[1] The AFib prevalence estimate for those aged ≥ 65 and with hypertension was calculated using the following formula: ((1.3% * 0.82)*0.84) / (40,267,984*0.782)

Secondary analysis

**Metric**	**Data**	**Source**
USA AFib prevalence (2010)	2.7 million	The American Heart Association (AHA) Heart Disease and Stroke Statistics - 2019 Update[5] and United States Preventative Task Force (USPTF)[6]
	6.1 million	

© 2020 O'Sullivan JW et al. *JAMA Network Open.*

USA AFib Prevalence (2010) in those aged ≥ 65 years	Using 2.7 million estimate (0.82*2.7 million) = 2,214,000	82% of AFib occurs in participants aged ≥ 65[3]
	Using 6.1 million estimate (0.82 *6.1million) = 5,002,000	
The prevalence of AFib in those aged over 65 and with hypertension.	(2.7m or 6.1m* 0.82)*0.84	As stated above, 82% of AFib occurs in those aged ≥ 65[3] and 84% of those with AFib have hypertension[14]
Total US population over the age of 65 and with a history of hypertension	40,267,984*0.782 = 31,489,564	As stated above and below there were 40,267,984 2 65 years in 2010 US census and 78.2% of people aged over 65 have hypertension.[1]
USA population	218 years = 234,564,071	The 2010 US census outlines these.[7]
	2 45 years = 121,757,429	
	2 65 years = 40,267,984	

© 2020 O'Sullivan JW et al. *JAMA Network Open.*

eAppendix 2. Sensitivity Analyses

We assessed the effect of including the one study which used an imperfect reference standard[7] in our analyses (this study used a chest belt to measure a one lead EKG as a reference standard). The inclusion of this study did not substantially, nor meaningfully alter the results from our primary analysis (appendix tables 5-8); the meta-analyzed sensitivity (for all apps collectively) did not change and the specificity decreased by 0.2%. Similarly, the meta-analyzed sensitivity and specificity for the Preventicus app did not substantially change (the study which used an imperfect reference standard[7] assessed the Preventicus app) : the meta-analyzed sensitivity increased by 0.5% and the meta-analysed specificity decreased by 0.8%. Lastly, the meta-regression confirmed the non-significant effect of the inclusion of an imperfect reference standard in our meta-analysis (the meta-regression coefficient and its corresponding 95%CI are reported in appendix table 6).

Our sensitivity analyses investigating the effect of verification bias on our results similarly showed non-meaningiful, tiny changes in meta-analyzed estimates of sensitivity, specificity and DOR. The meta-regression model also confirmed the non-significant effect of verification bias on our results (appendix tables 2, 5-7).

© 2020 O'Sullivan JW et al. *JAMA Network Open.*

Next, we conducted a sensitivity analysis to investigate the effect of the index and reference test not being applied concurrently. Two studies[8,9] did not state if the index and reference test were performed immediately after each other, or concurrently. Similar to the above sensitivity analyses, we found no significant effect on our results (appendix tables 2, 5-7).

Fourth, we assessed the effect of risk of bias on our results. As we did for the above analyses, we conducted a sensitivity analysis excluding studies that were rated as having a high risk of bias in at least one domain (five studies[7,8,10-12]). Again, there were no significant effect of high risk of bias on our results (appendix tables 2, 5-7).

Lastly, we investigated the difference in results between case-control designs and cohort designs. We observed no significant effect of the different designs in our meta-regression, nor did we observe a meaningful difference in meta-analyzed sensitivity, specificity, or DOR.

© 2020 O'Sullivan JW et al. *JAMA Network Open.*

eFigure 1. Study Flow Diagram

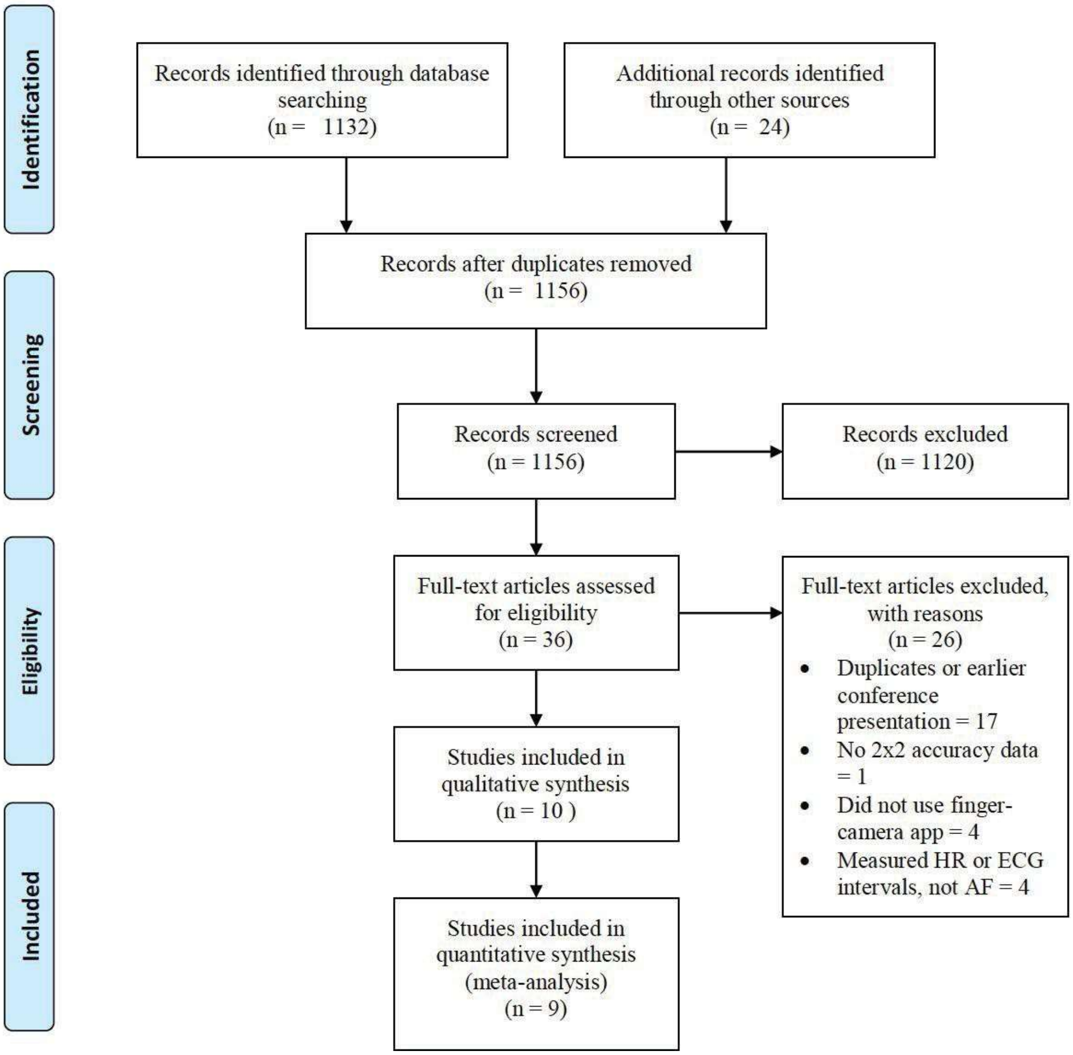

© 2020 O'Sullivan JW et al. *JAMA Network Open.*

eFigure 2. PPV and NPV of All Specific Applications Using Undiagnosed AF Prevalence Estimate of 1.3%

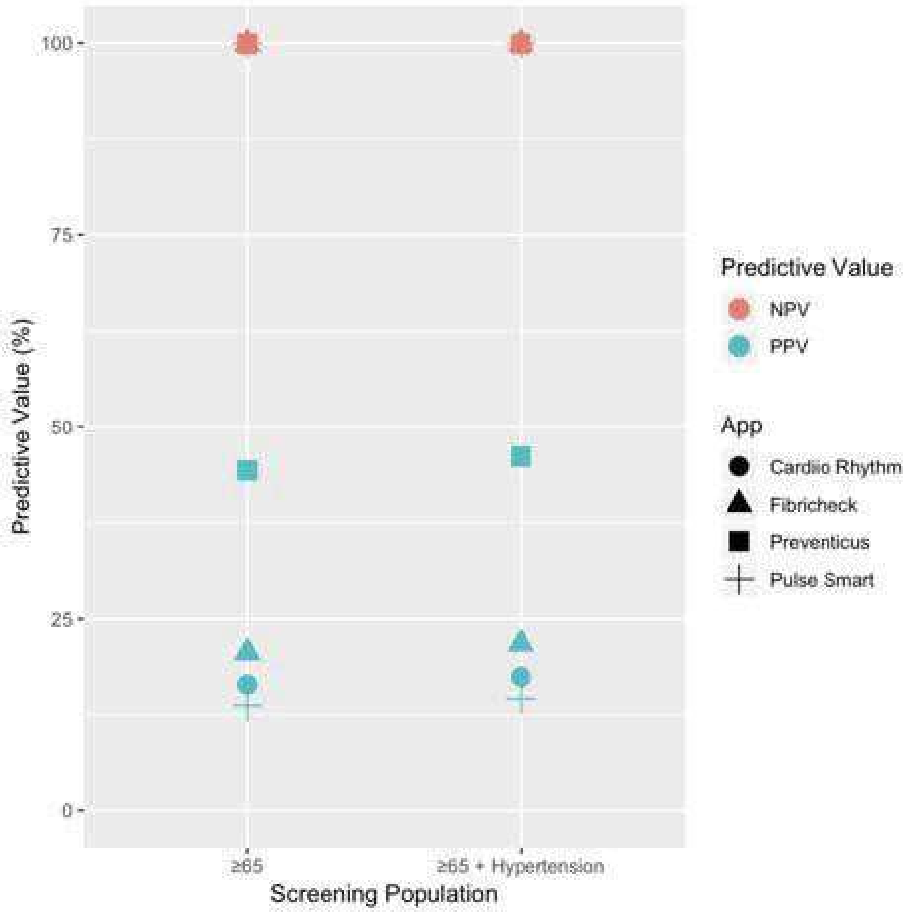

© 2020 O'Sullivan JW et al. *JAMA Network Open.*

eFigure 3. PPV and NPV of All Specific Applications Using Undiagnosed AF Prevalence Estimate of 1.3% and 3.2% Among Individuals Aged 65 Years and Older With Hypertension

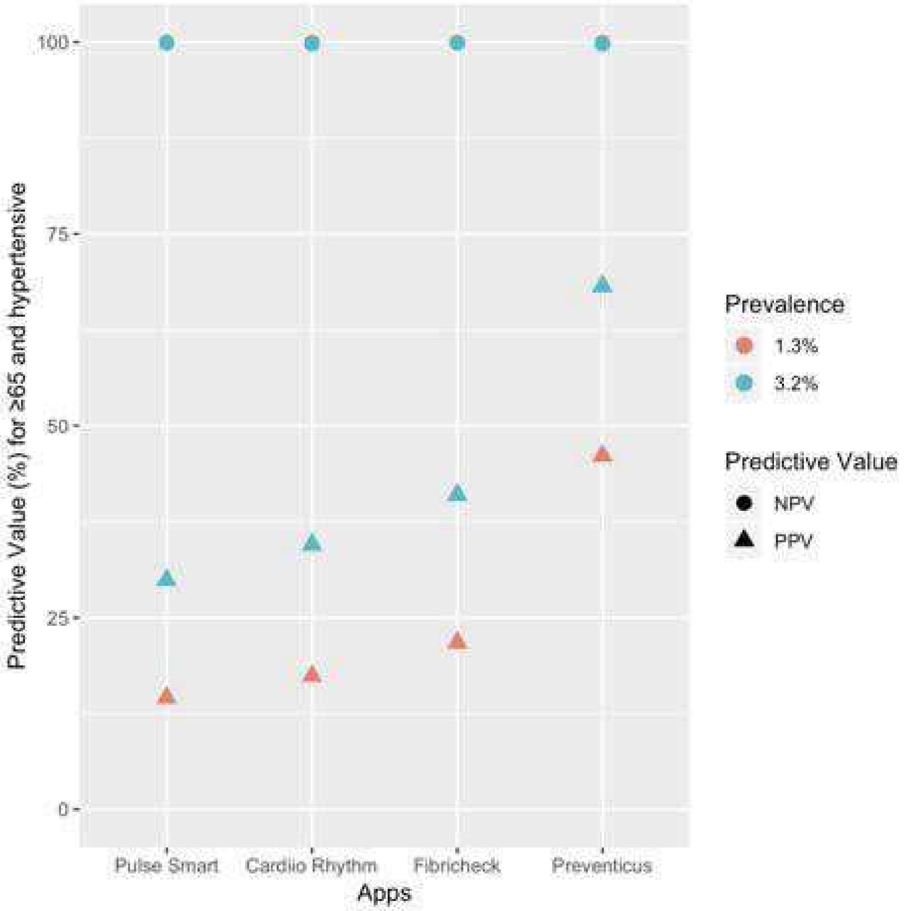

© 2020 O’Sullivan JW et al. *JAMA Network Open.*

eFigure 4. PPV and NPV for Each Application Using the AHA AF Prevalence Estimates

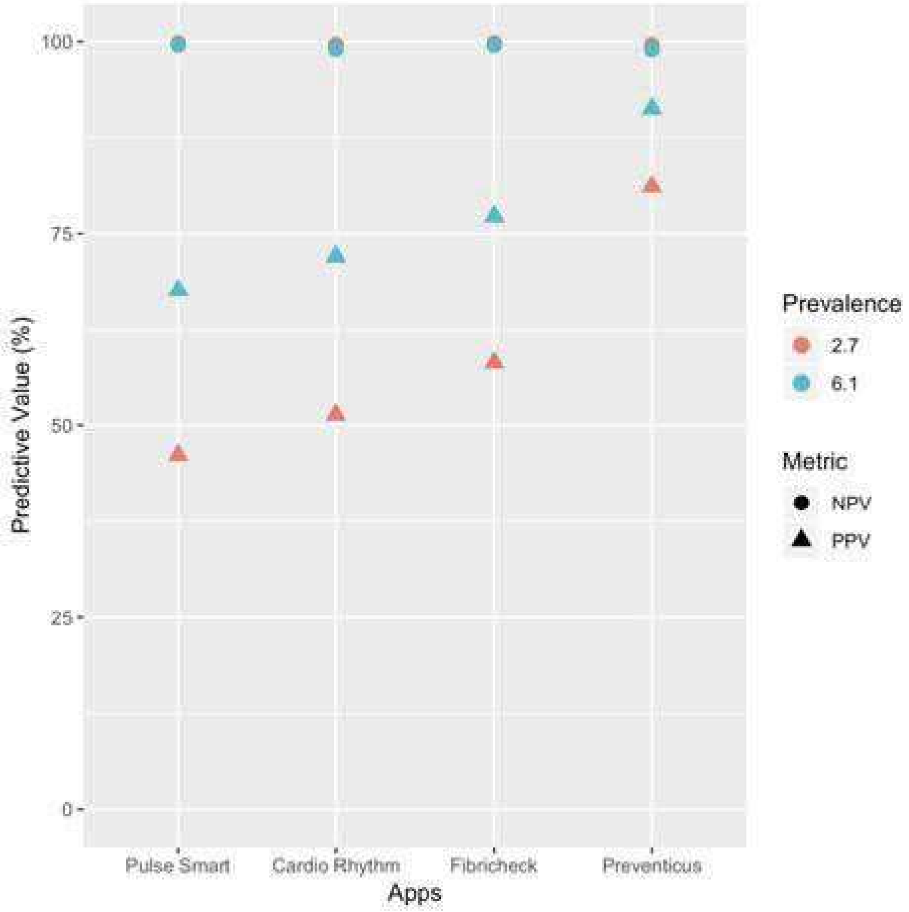

© 2020 O'Sullivan JW et al. *JAMA Network Open.*

eFigure 5. PPV for All Age Groups Using AHA AF Prevalence Estimates

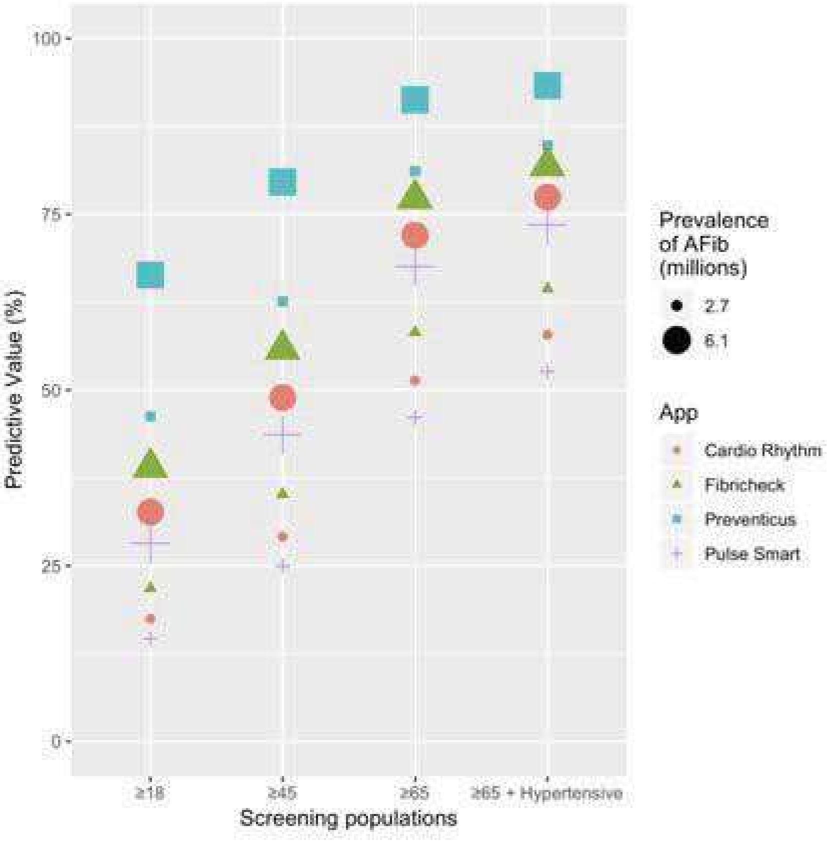

© 2020 O'Sullivan JW et al. *JAMA Network Open.*

eFigure 6. Summary Receiver Operating Characteristic Curve for Meta-analyzed Sensitivity and Specificity for All Applications Combined

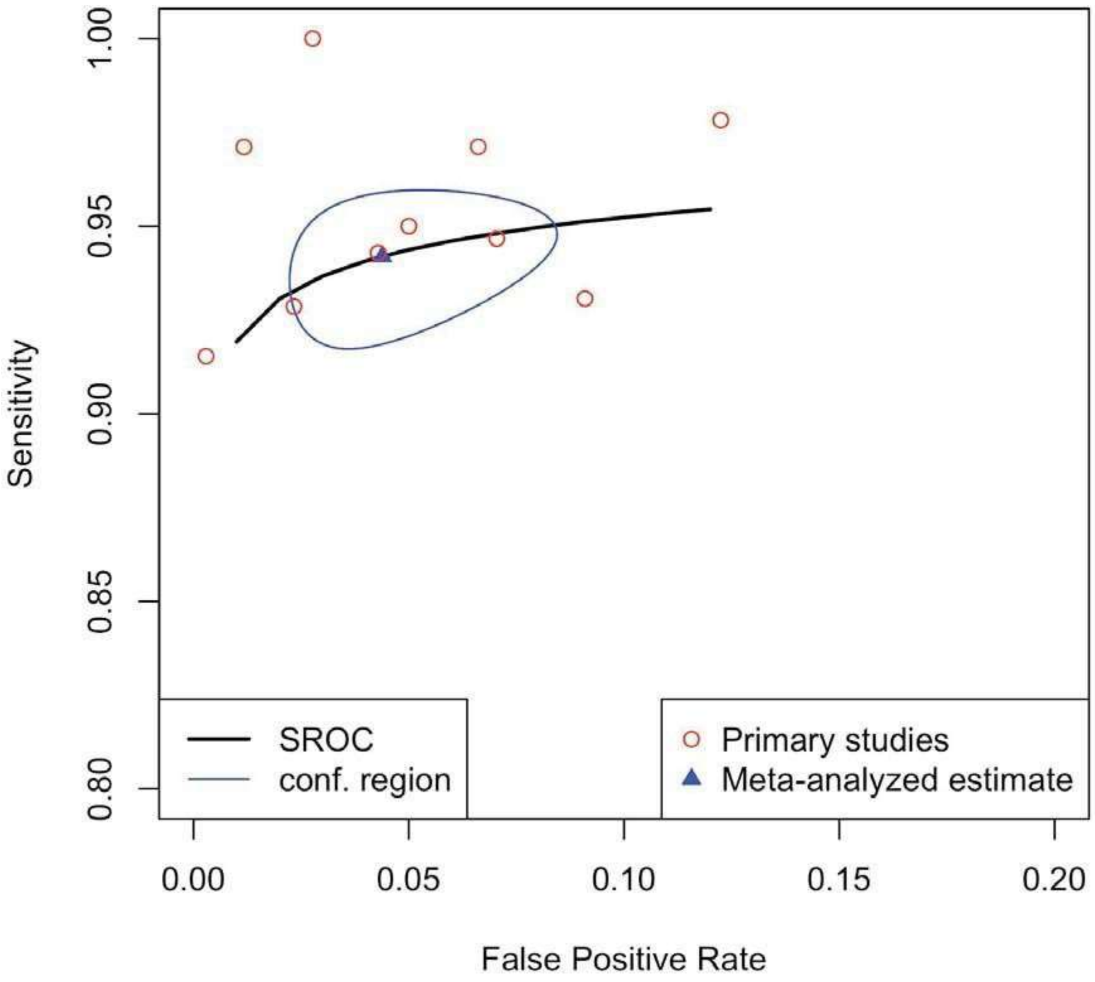

© 2020 O'Sullivan JW et al. *JAMA Network Open.*

eTable 1. Algorithm Details for Each Application

Study name	App	Algorithm	Algorithm methods paper reference
Brasier	Preventicus	This app used an algorithm called the "Heartbeats algorithm (Version 20171120)", which detects changes in time and morphology of PPG signals. Specifically it detects changes in the following parameters in the Time Domain: Standard deviation of the NN intervals (where NN interval is the RR interval, sdNN), Mean squared differences of consecutive NN intervals (rmssd), Rmssd normalized to the length of the tachogram (norm rmssd) and Shannon entropy ("Describes the variability of the observed values in bits") and the following parameters in the morphology Domain: Power in the low-frequency range; 0.04-0.15 Hz, Power in the high-frequency range; 0.15-0.4 Hz, Variance of all NN intervals; :S0.4 Hz, Normalized low-frequency power, Normalized high-frequency power.'	Koenig N et al. Validation of a New Heart Rate Measurement Algorithm for Fingertip Recording of Video Signals with Smartphones. Telemed J E Health. 2016 Aug;22(8):631-6
McManus	Pulsesmart	This app used an algorithm that had threshold values for time and morphology to classify a rhythm as sinus rhythm, AFib, or other (ectopic or non-sinus atrial and ventricular beats). The parameters they used to classify rhythm were: Root Mean Square of Successive Difference of RR intervals (RMSSD), Shannon Entropy (ShE), and Poincare plot (or Turning Point Ratio), using thresholds of: RMSDD = 0.1093, ShE = 0.4890, Poincare Plot = 0.2.	Dash S et al. Automatic real time detection of atrial fibrillation. Ann Biomed Eng. 2009 Sep;37(9):1701-9. Tateno K, Glass L. Automatic detection of atrial fibrillation using the coefficient of variation and density histograms of RR and deltaRR intervals. Med Biol Eng Comput. 2001 Nov;39(6):664-71.

© 2020 O'Sullivan JW et al. *JAMA Network Open.*

Krivoshei	Preventicus	This app used an algorithm that assessed variation in time and morphology of PPG signals: it assessed variation in Root mean square of successive difference of RR intervals (RMSSD), Shannon entropy (ShE), and SD1/SD2 (this ratio is taken from a Poincare plot, a Poincare plot plots RR interval against RR interval + 1, the SD1/SD2 ratio represents the variation ofdata).	Koenig N et al. Validation of a New Heart Rate Measurement Algorithm for Fingertip Recording of Video Signals with Smartphones. Telemed J E Health. 2016 Aug;22(8):631-6
Rozen	Cardiio Rhythm	This app used a "a supervised machine learning technique" to classify PPG signals. This supervised machine learning algorithm is known as "support vector machine"; they state this algorithm uses feature extraction to assess the "degree of self-similarity of a PPG waveform", but do not state further the underlying methodology, nor give a further reference	Not stated
Yan	Cardiio Rhythm	This app assessed repeated patterns in time and morphology of PPG waveforms and "classified the patterns using a previously trained support vector machine."	Not stated
Chan	Cardiio Rhythm	This app assessed the pattern of PPG waveforms, a lack of repeating pattern led to a diagnosis of AFib. Previously trained Support Vector Machine were used to classify patterns as non-repeating (AFib) or repeating (non-AF).	Not stated
Grieten	Fibricheck	Not stated	Not stated
Karim	Preventicus	Not stated	Not stated
Vandenberk	Fibricheck	Not stated	Not stated
Mortelmans	Fibricheck	The algorithm methodology is not stated in full, all that is stated is: "The raw single-lead signal quality was also scored by the filter software of the FibriCheck app (0: poor signal, unreliable result; 1: good signal, reliable result). QRS-complexes were detected using the Pan-Tompkins method"	Pan J, Tompkins WJ. A real-time QRS detection algorithm. IEEE Trans Biomed Eng. 1985, Mar; 32(3):230-6

To reach an AF diagnosis, these smartphone camera apps obtain a PPG signal from a user's fingertip pulse via a smartphone camera. The regularity of this PPG signal is then analyzed, both in terms of its morphology and its timing. A diagnosis of AF is made if the PPG signal reaches a threshold of irregular timing and a consecutive period of non-identical morphology (typically >30 seconds, measured in Hz) is observed. The irregularity of PPG timing is typically measured by Root Mean Square of Successive Difference of RR intervals (RMSSD), Shannon Entropy (ShE), and Poincare plots. The RMSSD

© 2020 O'Sullivan JW et al. *JAMA Network Open.*

represents beat-to-beat variation in heart rate and is obtained by first measuring the time difference between successive heartbeats (in ms). Then, the square of each value is calculated, averaged and then the square root of the total is calculated.[13] Shannon entropy is a statistical quantification of the probability of a random variable being observed. It is expressed on a scale of 0 to 1, where 1 implies the probability ofa random variable being observed is consistent e.g. a person's heart rate is at a regular, consistent rate. A result <1 implies a less consistent probability - i.e. a irregular heart rate (and this becomes less consistent as 0 is approached.[14] Lastly, Poincare plot shows the RR interval against RR interval + 1 and provides a visual description of the variation in RR interval plots. A SD1/SD2 ratio can be extracted from this plot and can quantify the variation in data.[7] The smartphone camera apps in our included studies measured the pulse for on average 2 minutes (range 1-5 minutes).

© 2020 O'Sullivan JW et al. *JAMA Network Open.*

eTable 2. Extended Characteristics of Included Studies

**Study name**	**App**	**Device**	**Time length of index test**	**Average Age**	**Percentage female**	**No. with AF (%)**	**No. with hypertension (%)**
Brasier	Preventicus	iPhone 4S	5 mins	Median: 78 (interquartile range 13)	45%	248 (42%)	427 (72.1%) in total, 241 (70.1%) in SR group, 186 (75%) in AFib group
McManus	Pulsesmart	iPhone 4S	2 mins	Mean: 65.9 (SD: 12.2) in AF group, mean 66 (SD: 11.9) in SR group	18%	104 (86%)	70 (71.4%) in the AF group, 63 (69.2%) in the SR group
Krivoshei	Preventicus	iPhone 4S	5 mins	Mean: 80 (SD: 8) in AF group, mean 75 in SR group (SD: 7)	30% in the AF group, 27.5% in the SR group	40 (50%)	Not stated
Rozen	Cardiio Rhythm	iPhone (generation not stated)	3x20 seconds	Mean: 67.7 (SD: 10.5)	25%	96 (98%)	Not stated
Yan	Cardiio Rhythm	iPhone 6S	3x20 seconds	Mean: 70.3 (SD: 13.9) in total. Mean in AF group: 75 (SD: 10), mean in non-AF group: 67.8 (SD: 15)	29%	75 (35%)	130 (59.9%) in total, 53 (70.7%) in AF group, 77 (54.2%) in non-AF group

© 2020 O'Sullivan JW et al. *JAMA Network Open.*

Chan	Cardiio Rhythm	iPhone 4S	3x17.1 seconds	Mean: 68.4 (SD: 12.2)	53%	28 (2.8%)	916 (90.4%) in total
Grieten	Fibricheck	Not stated	60 seconds	Mean: 59 (SD: 15)	59%	8 (0.8%)	Not stated
Karim	Preventicus	Not stated	Not stated	Mean: 74 (SD: 12) in AF group, mean: 60 (SD: 20) in the SR group	40% in the AF group, 34% in the SR group	70 (50%)	Not stated
Vandenberk	Fibricheck	Not stated	60 seconds	Not stated	Not stated	173 (50%)	Not stated
Mortelmans	Fibricheck	iPhone 5S	3x60 seconds	Mean: 78 (SD: 8) in total, 80 (SD: 8) in AF group, 76 (SD: 8) in non-AF group	51.5% in total, 47.7% in AF group, 43% in non-AF group	92 (48%)	198 (83.5%) in total, 102 (92%) in AF group, 96 (76.2%) in non-AF group

© 2020 O'Sullivan JW et al. *JAMA Network Open.*

eTable 3. Diagnostic Odds Ratio

**Analysis**	**Diagnostic Odds Ratio (DOR) (95%CT)**	**Logged DOR (95%CT)**
Primary: All studies except those with imperfect reference standard (Krivoshei)	400.5 (204.8 to 783.2)	6.0 (5.3 to 6.7)
Sensitivity analysis 1: All studies	380.6 (205.6 to 704.6)	5.9 (5.3 to 6.6)
Sensitivity analysis 2: Exclude studies with verification bias	508.2 (215.3 to 1199.5)	6.2 (5.4 to 7.1)
Sensitivity analysis 3: Exclude studies with non-immediate/simultaneous index and reference timing	332.4 (166.5 to 663.8)	5.8 (5.1 to 6.5)
Sensitivity analysis 4: Case-control design only	404.5 (156.0 to 1048.7)	6.0 (5.1 to 7.0)
Sensitivity analysis 4: Cohort design only	523.3 (175.8 to 1557.5)	6.3 (5.2 to 7.4)
Sensitivity analysis 5: Exclude studies with at least one domain high RoB	363.7 (172.5 to 766.9)	5.9 (5.2 to 6.6)
Sensitivity analysis 6: Exclude conference abstracts and theses	401.7 (157.3 to 1025.6)	6.0 (5.1 to 7.0)

*All Sensitivity analyses (after Sensitivity analysis 1) have Krivoshei excluded

© 2020 O'Sullivan JW et al. *JAMA Network Open.*

eTable 4. PPV and NPV

4.1 Primary analysis PPV and NPV (All apps, using undiagnosed AFib prevalence estimates)

**Age**	**Prevalence**	**Predictive Value**	**Value (%)**
≥65	1.30%	PPV	19.4
≥65	1.30%	NPV	99.9
≥65	3.20%	PPV	37.5
≥65	3.20%	NPV	99.8
≥65 + Hypertensive	1.30%	PPV	20.5
≥65 + Hypertensive	1.30%	NPV	99.9
≥65 + Hypertensive	3.20%	PPV	39.2
≥65 + Hypertensive	3.20%	NPV	99.8

© 2020 O’Sullivan JW et al. *JAMA Network Open.*

Appendix table 4.2 Primary analysis PPV and NPV (Specific apps, using undiagnosed AFib prevalence estimates)

**Age**	**Prevalence**	**Predictive Value**	**App**	**Value (%)**
≥65	1.30%	PPV	Preventicus	44.3
≥65	1.30%	NPV	Preventicus	99.9
≥65	1.30%	PPV	Pulse Smart	13.7
≥65	1.30%	NPV	Pulse Smart	99.9
≥65	1.30%	PPV	Fibricheck	20.5
≥65	1.30%	NPV	Fibricheck	99.9
≥65	1.30%	PPV	Cardiio Rhythm	16.4
≥65	1.30%	NPV	Cardiio Rhythm	99.9
≥65	3.20%	PPV	Preventicus	66.6
≥65	3.20%	NPV	Preventicus	99.8
≥65	3.20%	PPV	Pulse Smart	28.4
≥65	3.20%	NPV	Pulse Smart	99.9

© 2020 O'Sullivan JW et al. *JAMA Network Open.*

≥65	3.20%	PPV	Fibricheck	39.2
≥65	3.20%	NPV	Fibricheck	99.9
≥65	3.20%	PPV	Cardiio Rhythm	32.8
≥65	3.20%	NPV	Cardiio Rhythm	99.8
≥65 + Hypertension	1.30%	PPV	Preventicus	46.1
≥65 + Hypertension	1.30%	NPV	Preventicus	99.9
≥65 + Hypertension	1.30%	PPV	Pulse Smart	14.5
≥65 + Hypertension	1.30%	NPV	Pulse Smart	99.9
≥65 + Hypertension	1.30%	PPV	Fibricheck	21.7
≥65 + Hypertension	1.30%	NPV	Fibricheck	99.9
≥65 + Hypertension	1.30%	PPV	Cardiio Rhythm	17.4
≥65 + Hypertension	1.30%	NPV	Cardiio Rhythm	99.9
≥65 + Hypertension	3.20%	PPV	Preventicus	68.2

© 2020 O'Sullivan JW et al. *JAMA Network Open.*

≥65 + Hypertension	3.20%	NPV	Preventicus	99.8
≥65 + Hypertension	3.20%	PPV	Pulse Smart	29.9
≥65 + Hypertension	3.20%	NPV	Pulse Smart	99.9
≥65 + Hypertension	3.20%	PPV	Cardiio Rhythm	34.5
≥65+ Hypertension	3.20%	NPV	Cardiio Rhythm	99.8
≥65 + Hypertension	3.20%	PPV	Fibricheck	41.0
≥65 + Hypertension	3.20%	NPV	Fibricheck	99.9

Appendix table 4.3: Secondary analysis PPV and NPV (All apps, using AHA prevalence estimate)

**Age**	**Prevalence (million)**	**PPV**	**NPV**
≥18	2.7	20.6%	99.9%
≥45	2.7	33.6%	99.9%
≥65	2.7	56.4%	99.7%
≥65 + HTN	2.7	58.3%	99.6%
≥18	6.1	37.3%	99.8%
≥45	6.1	54%	99.7%
≥65	6.1	76%	99.2%
≥65 + HTN	6.1	77.4%	99.1%

© 2020 O’Sullivan JW et al. *JAMA Network Open.*

Appendix table 4.4: PPV and NPV (Specific apps) using AHA AF prevalence (not just undiagnosed AF)

App	Prevalence (millions)	Age	PPV (%)	NPV (%)
Preventicus	2.7	≥18	46.2	99.9
Pulse Smart	2.7	≥18	14.6	99.9
Cardio Rhythm	2.7	≥18	17.4	99.9
Fibricheck	2.7	≥18	21.8	99.9
Preventicus	2.7	≥45	62.6	99.8
Pulse Smart	2.7	≥45	25.0	99.9
Cardio Rhythm	2.7	≥45	29.2	99.8
Fibricheck	2.7	≥45	35.2	99.9
Preventicus	2.7	≥65 + Hypertensive	82.3	99.5
Pulse Smart	2.7	≥65 + Hypertensive	48.0	99.8
Cardio Rhythm	2.7	≥65 + Hypertensive	53.2	99.6
Fibricheck	2.7	≥65 + Hypertensive	60.0	99.8
Preventicus	2.7	≥65	81.1	99.6
Pulse Smart	2.7	≥65	46.079907	99.8
Cardio Rhythm	2.7	≥65	51.351785	99.6
Fibricheck	2.7	≥65	58.2096576	99.8
Preventicus	6.1	≥18	66.3492157	99.8
Pulse Smart	6.1	≥18	28.1706669	99.9
Cardio Rhythm	6.1	≥18	32.6336686	99.8
Fibricheck	6.1	≥18	38.9955489	99.9
Preventicus	6.1	≥45	79.5701498	99.6
Pulse Smart	6.1	≥45	43.652796	99.8

© 2020 O'Sullivan JW et al. *JAMA Network Open.*

Cardio Rhythm	6.1	≥45	48.8988158	99.6
Fibricheck	6.1	≥45	55.8048799	99.8
Preventicus	6.1	≥65 + Hypertensive	91.9	98.9
Pulse Smart	6.1	≥65 + Hypertensive	69.3	99.5
Cardio Rhythm	6.1	≥65 + Hypertensive	73.6	99.0
Fibricheck	6.1	≥65 + Hypertensive	78.6	99.5
Preventicus	6.1	≥65	91.2847079	98.9
Pulse Smart	6.1	≥65	67.568195	99.6
Cardio Rhythm	6.1	≥65	72.0150509	99.0
Fibricheck	6.1	≥65	77.2504957	99.5

© 2020 O'Sullivan JW et al. *JAMA Network Open.*

eTable 5. Full QUADAS-2 Assessment

**Study author name**	Consecutive or random sample	Case-control avoided?	Avoid inappropriate exclusions?	RoB	Index test without results of ref	If threshold, pre-specified?	RoB	Ref standard correctly classify	Ref test without results of index	RoB	Interval between index and ref?	All patients get a ref	Did patients get same ref	All patients in analysis?	RoB
Brasier	No	No	Yes	High	Yes	Yes	Low	Yes	Yes	Low	Unclear	Yes	Yes	No	High
McManus	No	Unclear	Unclear	Unclear	Unclear	Yes	Low	Yes	Unclear	Low	Yes	Yes	Yes	No	Unclear
Krivoshei	Yes	No	Yes	High	Unclear	Yes	Low	Unclear	Unclear	High	Unclear	Yes	Yes	Yes	Low
Rozen	Yes	No	Yes	Unclear	No	Yes	Low	Yes	No	Unclear	Yes	Yes	Yes	No	Low
Yan	Unclear	Yes	Yes	Unclear	Yes	Yes	Low	Yes	Yes	Low	Yes	Yes	Yes	No	Low
Chan	Unclear	Yes	Unclear	Unclear	Yes	Yes	Low	Yes	Yes	Low	Yes	No	Yes	No	High
Grieten	Unclear	Unclear	Unclear	Unclear	Unclear	Yes	Low	Yes	Unclear	Unclear	Unclear	No	Yes	Yes	High
Karim	Unclear	Yes	Yes	Unclear	Unclear	Yes	Low	Yes	Unclear	Unclear	Unclear	Yes	Yes	Yes	Low
Vandenberk	No	Unclear	Unclear	Unclear	Yes	Yes	Low	Yes	Yes	Low	Yes	Yes	Yes	Yes	Low
Mortelmans	No	No	Yes	High	Unclear	Yes	Low	Yes	Yes	Low	Yes	Yes	Yes	No	High

© 2020 O'Sullivan JW et al. *JAMA Network Open.*

eTable 6. Meta-analyzed Sensitivities and Specificities

Analysis	Sensitivity (95%CT)	Specificity (95%CT)
Primary: All studies except those with imperfect reference standard (Krivoshei)	94.2% (92.2% to 95.7%)	95.8% (92.4% to 97.7%)
Sensitivity analysis 1: All studies	94.2% (92.3% to 95.7%)	95.6% (92.6% to 97.4%)
Sensitivity analysis 2: Exclude studies with verification bias	94.8% (92.6% to 96.4%)	96.0% (90.9% to 98.3%)
Sensitivity analysis 3: Exclude studies with non-immediate/simultaneous index and reference timing	95.2% (93.0% to 96.7%)	95.0% (91.2% to 97.2%)
Sensitivity analysis 4: Case-control design only	94.5% (91.7% to 96.4%)	95.0% (87.0% to 98.2%)
Sensitivity analysis 4: Cohort design only	95.2% (91.6% to 97.3%)	97.0% (94.4% to 98.4%)
Sensitivity analysis 5: Exclude studies with at least one domain high RoB	95.3% (89.6% to 98.0%)	95.1% (89.6% to 97.8%)
Sensitivity analysis 6: Conference abstracts and theses excluded	93.7% (90.9% to 95.6%)	96.1% (90.4% to 98.5%)

© 2020 O'Sullivan JW et al. *JAMA Network Open.*

eTable 7. Metaregression

Covariate	Sensitivity P value	False positive rate (1-specificity) P value
Imperfect reference standard	0.9	0.7
Verification bias	0.6	0.3
Non-current index and reference test	0.06	0.2
Cohort study design	0.7	0.3
High risk of bias in at least one domain	0.9	0.5
Conference abstracts and theses excluded	0.1	0.9

eTable 8. Meta-analyzed Sensitivity and Specificity for Individual Applications

App	Analysis	Sensitivity (95%CT)	Specificity (95%CT)
Preventicus	Primary: All studies except those with imperfect reference standard (Krivoshei)	92.9% (88.1% to 95.8%)	98.7% (84.3% to 99.9%)
	Sensitivity analysis 1: All studies	93.4% (89.6% to 95.9%)	97.9% (89.3% to 99.6%)
	Sensitivity analysis 2: Exclude studies with verification bias	92.9% (88.1% to 95.8%)	98.7% (84.3% to 99.9%)
	Sensitivity analysis 3: Exclude studies with non-immediate/simultaneous index and reference timing	N/At	N/At
Pulse Waveform	Primary: All studies except those with imperfect reference standard (Krivoshei)	97.1% (91.4% to 99.1%)*	93.4% (87.3% to 96.7%)*
	Sensitivity analysis 1: All studies	Same as primary analysis	Same as primary analysis
	Sensitivity analysis 2: Exclude studies with verification bias	Same as primary analysis	Same as primary analysis
	Sensitivity analysis 3: Exclude studies with non-immediate/simultaneous index and reference timing	Same as primary analysis	Same as primary analysis
Cardiio Rhythm Mobile	Primary: All studies except those with imperfect reference standard (Krivoshei)	93.5% (89.2% to 96.2%)	94.8% (88.3% to 97.8%)
	Sensitivity analysis 1: All studies	Same as primary analysis	Same as primary analysis
	Sensitivity analysis 2: Exclude studies with verification bias	93.7% (89.0% to 96.5%)	92.1% (87.8% to 95.0%)
	Sensitivity analysis 3: Exclude studies with non-immediate/simultaneous index and reference timing	93.5% (89.2% to 96.2%)	94.8% (88.3% to 97.8%)
Fibricheck	Primary: All studies except those with imperfect reference standard (Krivoshei)	96.9% (94.1% to 98.4%)	96.0% (86.6% to 98.9%)

© 2020 O'Sullivan JW et al. *JAMA Network Open.*

	Sensitivity analysis 1: All studies	Same as primary analysis	Same as primary analysis
	Sensitivity analysis 2: Exclude studies with verification bias	97.5% (94.6% to 98.9%)	95.8% (67.7% to 99.6%)
	Sensitivity analysis 3: Exclude studies with non-immediate/simultaneous index and reference timing	96.9% (94.1% to 98.4%)	96.0% (86.6% to 98.9%)

*There is only one study for Pulse Waveform so this is not meta-analyzed
t All studies referring to this app excluded for this analysis

© 2020 O'Sullivan JW et al. *JAMA Network Open.*

eReferences

1. Turakhia MP, Shafrin J, Bognar K, et al. Estimated prevalence of undiagnosed atrial fibrillation in the United States. *PLoS One*. 2018;13(4):e0195088.

2. Apple Heart Study Identifies AFib in Small Group of Apple Watch Wearers - American College of Cardiology. American College of Cardiology. https://www.acc.org/latest-in-cardiology/articles/2019/03/08/15/32/sat-9am-apple-heart-study-acc-2019. Accessed March 26, 2019.

3. Go AS, Hylek EM, Phillips KA, et al. Prevalence of diagnosed atrial fibrillation in adults: national implications for rhythm management and stroke prevention: the AnTicoagulation and Risk Factors in Atrial Fibrillation (ATRIA) Study. *JAMA*. 2001;285(18):2370-2375.

4. Piccini JP, Hammill BG, Sinner MF, et al. Incidence and Prevalence of Atrial Fibrillation and Associated Mortality Among Medicare Beneficiaries: 1993-2007. *Circulation: Cardiovascular Quality and Outcomes*. 2012;5(1):85-93. doi:10.1161/circoutcomes.111.962688

5. Benjamin EJ, Muntner P, Alonso A, et al. Heart Disease and Stroke Statistics-2019 Update: A Report From the American Heart Association. *Circulation*. 2019;139(10):e56-e66.

6. US Preventive Services Task Force, Curry SJ, Krist AH, et al. Screening for Atrial Fibrillation With Electrocardiography: US Preventive Services Task Force Recommendation Statement. *JAMA*. 2018;320(5):478-484.

7. Krivoshei L, Weber S, Burkard T, et al. Smart detection of atrial fibrillationt. *Europace*. 2017;19(5):753-757.

8. Brasier N, Raichle CJ, Dorr M, et al. Detection of atrial fibrillation with a smartphone camera: first prospective, international, two-centre, clinical validation study (DETECT AF PRO). *Europace*. 2019;21(1):41-47.

9. Karim N, Aral M, Eduawor S, Finlay M. AF detection using smartphone apps. *Europace*. 2017;19 (Supplement 3):iii234.

10. Mortelmans C. Validation of a new smartphone application ("FibriCheck") for the diagnosis of atrial fibrillation in primary care. Vaes B, ed. 2015.

© 2020 O'Sullivan JW et al. *JAMA Network Open.*

https://www.scriptieprijs.be/sites/default/files/thesis/2016-10/Manama-thesis%20Mortelmans%20Christophe%20-%20Validatiestudie%20VKF.pdf.

11. Chan P-H, Wong C-K, Poh YC, et al. Diagnostic Performance of a Smartphone-Based Photoplethysmographic Application for Atrial Fibrillation Screening in a Primary Care Setting. *J Am Heart Assoc*. 2016;5(7). doi:10.1161/JAHA.116.003428

12. Grieten L, Van Der Auwera J, Vandervoort P, Dujardin K. Using smartphone enabled technologies for detection atrial fibrillation: Is there a difference in signal quality between ECG and PPG? (Conference abstract). *Heart Rhythm*. 2018;15(5):Supplement 1 (S70-S71).

13. Shaffer F, Ginsberg JP. An Overview of Heart Rate Variability Metrics and Norms. *Front Public Health*. 2017;5:258.

14. Pathria RK, Beale PD. *Statistical Mechanics*. Butterworth-Heinemann; 2011.

© 2020 O'Sullivan JW et al. *JAMA Network Open.*
